# Supplementary figures and images for: Current ionising radiation doses in the Chernobyl Exclusion Zone do not directly impact on soil biological activity
Source: PLoS One. 2022 Feb 23;17(2):e0263600. doi: 10.1371/journal.pone.0263600 (PMC8865656; doi:10.1371/journal.pone.0263600)

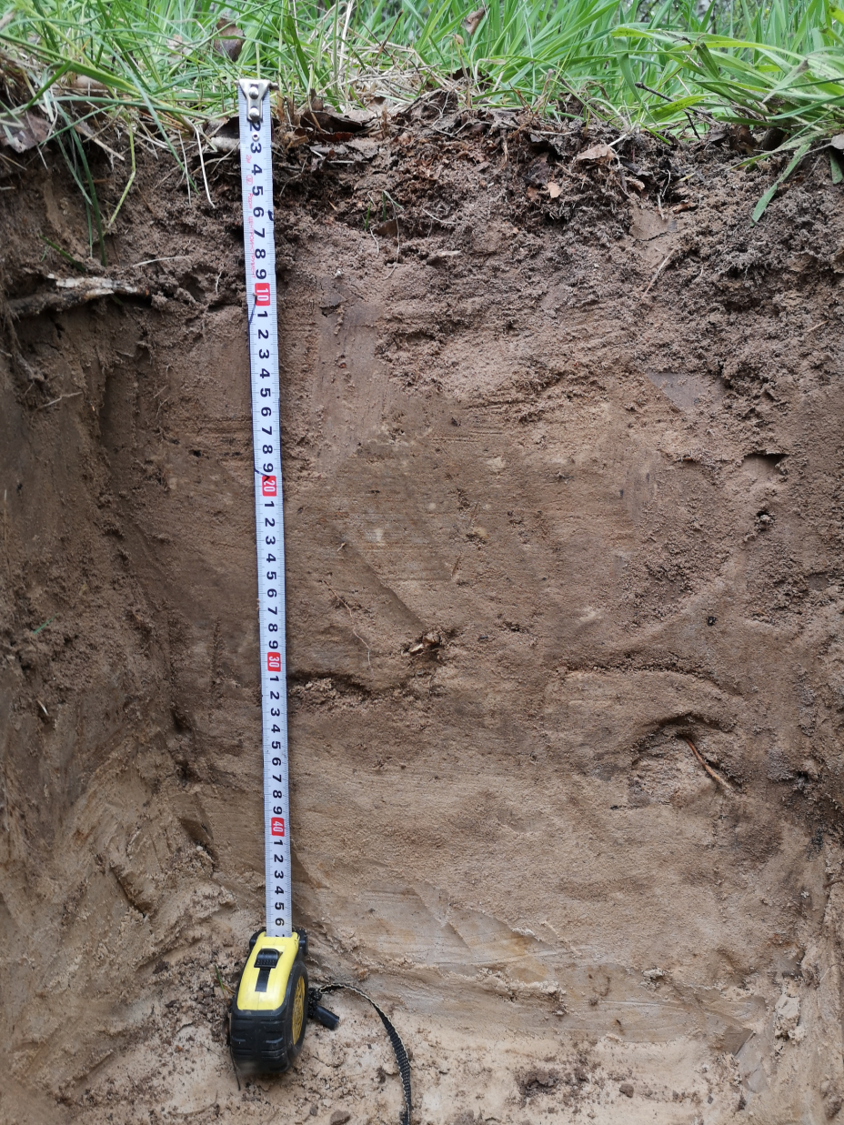

Supplement: S1 Fig — (TIF) [file pone.0263600.s001.tif]

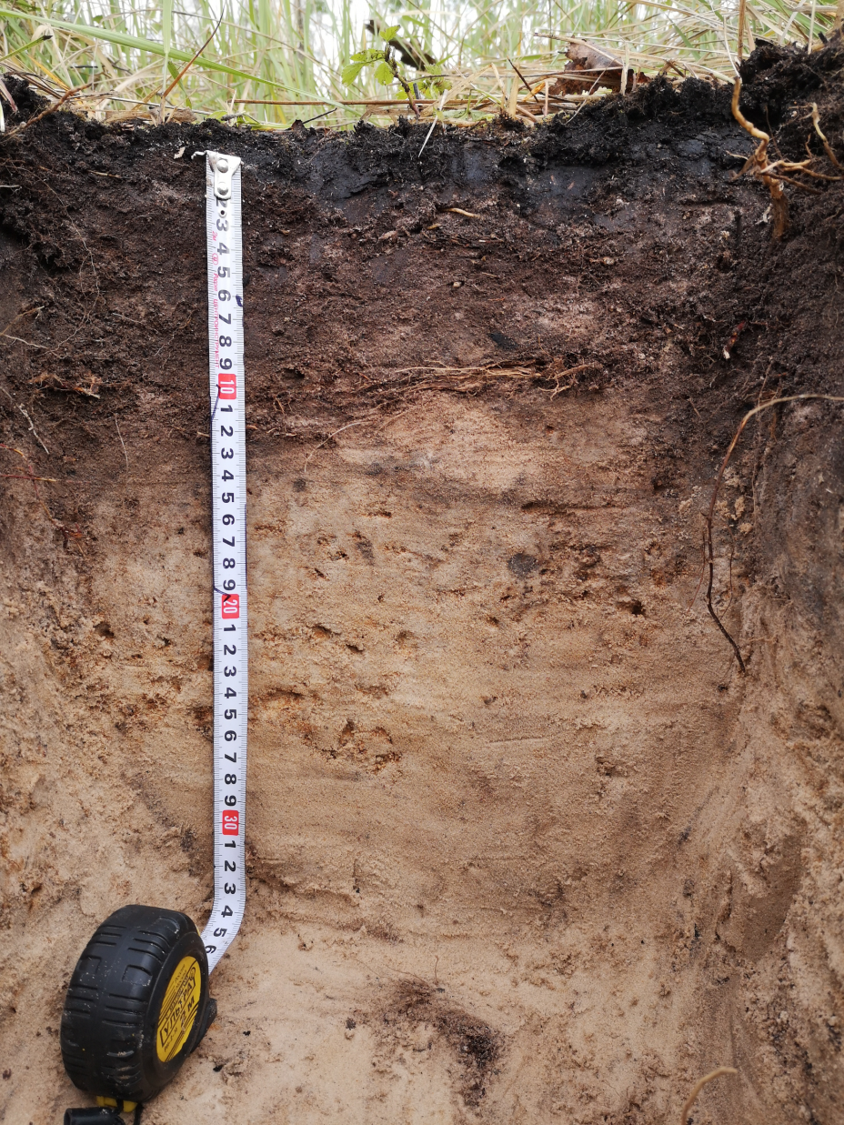

Supplement: S2 Fig — (TIF) [file pone.0263600.s002.tif]
