# Supplementary material for: Current ionising radiation doses in the Chernobyl Exclusion Zone do not directly impact on soil biological activity
Source: PLoS One. 2022 Feb 23;17(2):e0263600. doi: 10.1371/journal.pone.0263600 (PMC8865656; doi:10.1371/journal.pone.0263600)
Supplement: Attachment — Submitted filename: PONE-D-21-24695_reviewer-MB.pdf [file pone.0263600.s003.pdf]

PLOS ONE

Current ionising radiation doses in the Chernobyl Exclusion Zone do not directly impact on soil biological activity

--Manuscript Draft--

**Manuscript Number:**	PONE-D-21-24695
**Article Type:**	Research Article
**Full Title:**	Current ionising radiation doses in the Chernobyl Exclusion Zone do not directly impact on soil biological activity
**Short Title:**	Current ionising radiation doses in the Chernobyl Exclusion Zone - impact on soil biological activity
**Corresponding Author:**	Nick Beresford<br>UK Centre for Ecology & Hydrology<br>Lancaster, UNITED KINGDOM
**Keywords:**	Red Forest; bait lamina; absorbed dose; invertebrate; ecosystem function
**Abstract:**	Although soil organisms are essential for ecosystem function, the impacts of radiation on soil biological activity at highly contaminated sites has been relatively poorly studied. In April-May 2016, we conducted the first largescale deployment of bait lamina to estimate soil organism (largely soil invertebrate) feeding activity in situ at study plots in the Chernobyl Exclusion Zone (CEZ). Across our 53 study plots, estimated weighted absorbed dose rates to soil organisms ranged from 0.7 µGy h-1 to 1753 µGy h-1. There was no significant relationship between soil organism feeding activity and estimated weighted absorbed dose rate. Soil biological activity did show significant relationships with soil moisture content, bulk density (used as a proxy for soil organic matter) and pH. At plots in the Red Forest (an area of coniferous plantation where trees died because of high radiation exposure in 1986) soil biological activity was low compared to plots elsewhere in the CEZ. It is possible that the lower biological activity observed in the Red Forest is a residual consequence of what was in effect an acute high exposure to radiation in 1986.
**Order of Authors:**	Nicholas A. Beresford
	Michael D. Wood
	Sergey Gashchak
	Catherine L. Barnett
**Additional Information:**	
**Question**	**Response**
**Financial Disclosure**<br><br>Enter a financial disclosure statement that describes the sources of funding for the work included in this submission. Review the submission guidelines for detailed requirements. View published research articles from *PLOS ONE* for specific examples.<br><br>This statement is required for submission and **will appear in the published article** if the submission is accepted. Please make sure it is accurate.	The work described in this paper was conducted as part of the TREE project (www.ceh.ac.uk/TREE), which was funded under the RATE Programme (https://www2.bgs.ac.uk/rate/home.html) by the Natural Environment Research Council, the Environment Agency and Radioactive Waste Management Ltd. (grant codes: NE/L000318/1 and NE/L000520/1). All authors received funding via this route. The funders had no role in study design, data collection and analysis, decision to publish, or preparation of the manuscript.

Powered by Editorial Manager® and ProduXion Manager® from Aries Systems Corporation

**Unfunded studies**<br>Enter: *The author(s) received no specific funding for this work.*<br><br>**Funded studies**<br>Enter a statement with the following details:<br>• Initials of the authors who received each award<br>• Grant numbers awarded to each author<br>• The full name of each funder<br>• URL of each funder website<br>• Did the sponsors or funders play any role in the study design, data collection and analysis, decision to publish, or preparation of the manuscript?<br>• **NO** - Include this sentence at the end of your statement: *The funders had no role in study design, data collection and analysis, decision to publish, or preparation of the manuscript.*<br>• **YES** - Specify the role(s) played.<br><br>* typeset	
**Competing Interests**<br><br>Use the instructions below to enter a competing interest statement for this submission. On behalf of all authors, disclose any competing interests that could be perceived to bias this work—acknowledging all financial support and any other relevant financial or non-financial competing interests.<br><br>This statement is required for submission and **will appear in the published article** if the submission is accepted. Please make sure it is accurate and that any funding sources listed in your Funding Information later in the submission form are also declared in your Financial Disclosure statement.<br><br>View published research articles from *PLOS ONE* for specific examples.	The authors have declared that no competing interests exist.

**NO authors have competing interests**<br><br>Enter: *The authors have declared that no competing interests exist.*<br><br>**Authors with competing interests**<br><br>Enter competing interest details beginning with this statement:<br><br>*I have read the journal's policy and the authors of this manuscript have the following competing interests: [insert competing interests here]*<br><br>* typeset	
**Ethics Statement**<br><br>Enter an ethics statement for this submission. This statement is required if the study involved:<br><br>• Human participants<br>• Human specimens or tissue<br>• Vertebrate animals or cephalopods<br>• Vertebrate embryos or tissues<br>• Field research<br><br>Write "N/A" if the submission does not require an ethics statement.<br><br>General guidance is provided below. Consult the submission guidelines for detailed instructions. **Make sure that all information entered here is included in the Methods section of the manuscript.**	Permission for research activities and sampling within the Chernobyl Exclusion Zone was granted to Chornobyl Center by the State Agency of Ukraine for the Exclusion Zone Management.

Powered by Editorial Manager® and ProduXion Manager® from Aries Systems Corporation

**Format for specific study types**<br><br>**Human Subject Research (involving human participants and/or tissue)**<br>• Give the name of the institutional review board or ethics committee that approved the study<br>• Include the approval number and/or a statement indicating approval of this research<br>• Indicate the form of consent obtained (written/oral) or the reason that consent was not obtained (e.g. the data were analyzed anonymously)<br><br>**Animal Research (involving vertebrate animals, embryos or tissues)**<br>• Provide the name of the Institutional Animal Care and Use Committee (IACUC) or other relevant ethics board that reviewed the study protocol, and indicate whether they approved this research or granted a formal waiver of ethical approval<br>• Include an approval number if one was obtained<br>• If the study involved *non-human primates*, add *additional details* about animal welfare and steps taken to ameliorate suffering<br>• If anesthesia, euthanasia, or any kind of animal sacrifice is part of the study, include briefly which substances and/or methods were applied<br><br>**Field Research**<br><br>Include the following details if this study involves the collection of plant, animal, or other materials from a natural setting:<br>• Field permit number<br>• Name of the institution or relevant body that granted permission	
**Data Availability**<br><br>Authors are required to make all data underlying the findings described fully available, without restriction, and from the time of publication. PLOS allows rare exceptions to address legal and ethical concerns. See the PLOS Data Policy and FAQ for detailed information.	Yes - all data are fully available without restriction

A Data Availability Statement describing where the data can be found is required at submission. Your answers to this question constitute the Data Availability Statement and **will be published in the article**, if accepted.<br><br>**Important:** Stating 'data available on request from the author' is not sufficient. If your data are only available upon request, select 'No' for the first question and explain your exceptional situation in the text box.<br><br>Do the authors confirm that all data underlying the findings described in their manuscript are fully available without restriction?	
**Describe where the data may be found in full sentences. If you are copying our sample text, replace any instances of XXX with the appropriate details.**<br><br>• If the data are **held or will be held in a public repository**, include URLs, accession numbers or DOIs. If this information will only be available after acceptance, indicate this by ticking the box below. For example: *All XXX files are available from the XXX database (accession number(s) XXX, XXX.).*<br>• If the data are all contained **within the manuscript and/or Supporting Information files**, enter the following: *All relevant data are within the manuscript and its Supporting Information files.*<br>• If neither of these applies but you are able to provide **details of access elsewhere**, with or without limitations, please do so. For example:<br><br>*Data cannot be shared publicly because of [XXX]. Data are available from the XXX Institutional Data Access / Ethics Committee (contact via XXX) for researchers who meet the criteria for access to confidential data.*<br><br>*The data underlying the results presented in the study are available from (include the name of the third party*	NERC Environmental Information Data Centre. doi: 10.5285/19babe1c-b3a3-488c-b4fe-ebb4ab9237d8<br><br>Freely available now

and contact information or URL).<br>• This text is appropriate if the data are owned by a third party and authors do not have permission to share the data.<br><br>* typeset	
Additional data availability information:	

Powered by Editorial Manager® and ProduXion Manager® from Aries Systems Corporation

Manuscript

Click here to access/download;Manuscript;CEZ Bait Lamina April2016_final.docx

Current ionising radiation doses in the Chernobyl Exclusion Zone do not directly impact on soil biological activity

Nicholas A. Beresford[1,2*], Michael D. Wood[2], Sergey Gashchak[3], Catherine L. Barnett[1]

[1]UK Centre for Ecology & Hydrology, Lancaster Environment Centre, Bailrigg, Lancaster, United Kingdom

[2]School of Science, Engineering & Environment, University of Salford, Manchester, United Kingdom

[3]Chornobyl Center for Nuclear Safety, Radioactive Waste & Radioecology, International Radioecology Laboratory, Slavutych, Kyiv Region, Ukraine

[*] Corresponding author

Email: nab@ceh.ac.uk (NAB)

All authors contributed to this work equally.

Abstract

Although soil organisms are essential for ecosystem function, the impacts of radiation on soil biological activity at highly contaminated sites has been relatively poorly studied. In April-May 2016, we conducted the first largescale deployment of bait lamina to estimate soil organism (largely soil invertebrate) feeding activity *in situ* at study plots in the Chernobyl Exclusion Zone (CEZ). Across our 53 study plots, estimated weighted absorbed dose rates to soil organisms

ranged from 0.7 μGy h^{-1} to 1753 μGy h^{-1}. There was no significant relationship between soil organism feeding activity and estimated weighted absorbed dose rate. Soil biological activity did show significant relationships with soil moisture content, bulk density (used as a proxy for soil organic matter) and pH. At plots in the Red Forest (an area of coniferous plantation where trees died because of high radiation exposure in 1986) soil biological activity was low compared to plots elsewhere in the CEZ. It is possible that the lower biological activity observed in the Red Forest is a residual consequence of what was in effect an acute high exposure to radiation in 1986.

Introduction

Soil organisms are essential for ecosystem function, playing a vital role in processes such as organic matter decomposition, nutrient cycling (and availability to plants) and bioturbation [1]. The effect of various stressors, including pollutants, on soil biological activity has been the focus of much study [e.g. 2-8]. Although it has been recommended that soil fauna could be used as radiological biomonitors [9], the effects of ionising radiation on soil biological activity at radiologically contaminated field sites has been relatively poorly studied. This may, in part, be the consequence of soil organisms (invertebrate macrofauna, mesofauna and microorganisms) typically being thought to be relatively insensitive to radiation compared to other biota [10, 11]. Some studies have reported effects on soil fauna at sites with high levels of natural radionuclides, including uranium mines [12-15]. However, it is likely that chemical toxicity rather than radiation dose is the cause of effects observed at such sites. Three months after the 1986 Chernobyl nuclear power plant accident, a 30-fold reduction in forest litter organisms was recorded at a pine forest site 3 km to the south of the Chernobyl reactor (total absorbed dose of 29 Gy estimated from thermoluminescent dosimeters placed in soil) compared to a similar site 70 km south with an estimated dose about 40-fold lower [16, 17]. Within 2-2.5 years after the

accident, the biomass of soil fauna at the two sites was similar [16], but Geras'kin et al. [17] suggests that soil species diversity continued to be affected (approximately 20% reduction) 10 years after the Chernobyl accident.

In the longer-term after the accident, there is a lack of agreement in reported effects of radiation on soil organisms in the Chernobyl Exclusion Zone (CEZ); the CEZ is the approximately 4800 km^2 area around the Chernobyl Nuclear Power Plant (ChNPP) that was abandoned following the accident [18]. Mousseau et al. [19] deployed leaf litter bags at sites (forests or abandoned farmland reverting to forest) in the CEZ from September 2007 for nine months. Ambient dose rates ranged from 0.09 to 240 $\mu Sv\ h^{-1}$ (as determined by hand-held dosimeter) and the authors reported a significant reduction (by up to 40 %) in leaf litter decomposition with increasing radiation levels; accounting for potential confounding variables, such as pH and soil moisture, did not change their conclusion. The authors also stated that the lower leaf litter decomposition resulted in the accumulation of organic matter in areas with higher radiation levels. Conversely, Bonzom et al. [20] found no negative impact on litter decomposition measured using litter bags at deciduous and mixed forest sites with ambient dose rates ranging from 0.22 to 29 $\mu Gy\ h^{-1}$ (the authors estimated that this equated to a maximum absorbed dose rate for litter decomposers of 150 $\mu Gy\ h^{-1}$); litter bags were deployed in November 2011 for a total of 318 d. It should be acknowledged that the maximum ambient dose rate studied by Bonzom et al. is about an order of magnitude below that of Mousseau et al.. However, the data of Bonzom et al. suggested an increase in litter mass loss with increasing dose rate at dose rates where Mousseau et al.'s 'linear dose-response relationship' (*sic*) would predict a reduction in decomposition. Commenting on the contrasting findings of the Mousseau et al. [19] study and their own study, Bonzom et al. make the observation that the decomposition rates at some of the most contaminated sites of Mousseau et al. are comparable to, or higher than, those previously observed for litter from similar tree species at uncontaminated sites (see references cited by

Bonzom et al.). Other studies considering ecologically important soil organisms within the CEZ found little or no effect of radiation [21-24].

In this paper we evaluate soil biological activity at sites across an ambient dose rate gradient of 0.6 to 237 µSv h^{-1} during spring 2016 (estimated absorbed dose rates to soil organisms 0.7 to 1753 µGy h^{-1}). We used bait lamina [25] to measure soil organism feeding activity *in situ*. This approach has been used extensively to assess the impact of various chemical pollutants on soil biological activity [e.g. 3, 12, 26-28]. Bait lamina have been recommended as an indicator of soil biodiversity by a European Union working group [29] and as a method for use in ecological risk assessment [30]. Subsequently, the International Organization for Standardization (ISO) published ISO 18311:2016 [31], which defines a robust method for using bait lamina for field-determination of anthropogenic impacts on soil organism feeding activity.

All underlying data from the study reported here have been made openly available in a dataset published alongside this paper [32], enabling independent evaluation and reanalysis.

Materials and Methods

Study sites

Eighteen study sites were selected, all of which were located within the CEZ. Eight of the sites were located in the Red Forest, an area of about 4 – 6 km^2 to the west of the Chernobyl nuclear power plant where coniferous trees died as a consequence of high radiation exposure in the immediate aftermath of the accident [33]. At the time of this study, the area had regenerated with deciduous trees (predominantly *Betula* spp. (birch)) and generally sparse understorey vegetation including grasses, sedges, ericaceous species and, at a few sites, young pine saplings (site description notes can be found in the full dataset associated with this paper [32]). The decayed trunks of pine trees killed by the accident were also still evident on the ground surface. Within

the Red Forest it was possible to select study sites with an approximately twenty-fold variation in ambient dose rate; the ambient gamma dose rate (μSv h^{-1}) on the soil surface was measured at each study plot using a beta-shielded ATOMEX AT6130 dosimeter, the reading being taken once the meter had stabilised. The meter is calibrated annually and has a high precision (difference in measurements of <3% based on repeat readings at sites within the CEZ). Three sites were selected in mature mixed deciduous woodland to the south of the Red Forest (Fig 1) with ambient dose rates within the range of those in the Red Forest; at two of these sites there was evidence (i.e. dead mature pine trunks on the ground surface) suggesting that of the few pine trees previously present at these sites some had been killed by radiation following the accident. Two deciduous woodland sites were also selected to the west of the Red Forest (on the edge of the 'western trace' of the initial release from the 1986 accident) with a further two sites in an area of comparatively low deposition to the north-west of the Red Forest (Fig 1). The deciduous woodland sites to the south and north-west were visually different to the Red Forest, having a more substantial litter layer which was virtually absent at some of the Red Forest sites. The deciduous woodland sites to the west were more similar to the Red Forest with a general lack of a litter layer and sparse understorey vegetation (S1 and S2 Photographs). Three coniferous woodland sites were also selected in the area of comparatively low deposition (Fig 1). The locations of all study sites were recorded using a Garmin 64st Global Positioning System (GPS) with an accuracy of ±3 m.

Permission for research activities and sampling within the Chernobyl Exclusion Zone was granted to Chornobyl Center by the State Agency of Ukraine for the Exclusion Zone Management.

Fig 1. Location of sample sites within the Chernobyl Exclusion Zone; deposition decay corrected to 2016.

Bait lamina

The bait lamina strips were obtained from 'terra protecta GmbH' (http://www.terra-protecta.de/en/bait_strips.html). These semi-rigid polyvinyl chloride (PVC) strips (1 mm x 6 mm x 155 mm) have sixteen 1.5 mm apertures located at 5 mm intervals which begin 10 mm from the pointed lower end (the upper 70 mm of the strip has no apertures) [25, 31]. The apertures were filled with bait (for soil organisms) comprising 70% cellulose powder, 25% finely ground wheat bran and 5% activated charcoal.

Field application of the bait lamina followed ISO 18311:2016. At each of the sites, three 1x1 m plots with similar vegetation cover were identified; at a given site the plots were within c. 30 m of each other. At one of the deciduous woodland sites to the west of the Red Forest it was only possible to have two plots. Sixteen bait lamina strips were inserted into the ground within each plot (using a 4x4 grid with bait lamina approximately evenly spaced) during the period 17th-19th April 2016. To ensure that the bait lamina would not be damaged during insertion into the soil, a thin bladed knife of similar dimensions to the bait lamina strip was used to cut a channel into the soil. The strips were then inserted such that the top aperture was *c*. 0.5 cm below the soil surface. At each plot, an additional strip was inserted and withdrawn to determine whether soil abrasion would result in loss of bait from any of the 1.5 mm apertures. All bait lamina were retrieved after approximately 18 days (during 5th-7th May 2016). Soil temperature was measured in each plot at the time of bait lamina insertion and again at removal using a Eutech Instruments CyberScan pH300 (resolution 0.1 °C ± 0.3 °C).

After gentle wiping with tissue paper to remove adherent soil, the bait lamina sticks were visually assessed by placing them on an iPad tablet and using LightBox (v1.4) light table app. Feeding activity was assessed as qualitative feeding (i.e. the number of apertures showing any degree of bait consumption). A simple 'pierced' or 'unpierced' scoring system was used, where

pierced was defined as clear evidence of bait removal (removal being distinguished from cracking). The vertical distribution of evidence of feeding was recorded. The bait lamina sticks were each read blind by two people (i.e. the people reading the strips had no knowledge of where they had been deployed within the CEZ). For analyses and discussion, results for an individual plot have been summed across all 16 bait lamina strips.

Soil sampling and analyses

After removal of the bait lamina strips, five 10 cm deep soil cores (2.5 cm diameter) were collected from each plot (one from each corner and one from the middle). The five cores were bulked, homogenised and sub-samples taken and sealed in zip-lock plastic bags for subsequent determination of pH and percentage moisture. The remaining soil sample was dried at 60°C prior to radionuclide activity concentration determination. The pH of fresh soil was determined using the method of Allen [34] and percentage moisture was determined by mass loss when oven drying a sub-sample to constant mass at 60°C. Five separate soil samples were also collected from each plot using a 10 cm deep collector of 250 cm^3 volume, which was driven into the ground until it was level with the surface; this gave a soil sample of known volume which was subsequently oven dried at 60°C to enable the estimation of dry mass (DM) bulk density.

A Canberra-Packard gamma-spectrometer with a high-purity germanium (HPGe) detector (GC 3019) was used to determine the activity concentration of ^{137}Cs. For calibration a standard ^{44}Ti, ^{137}Cs and ^{152}Eu source comprising epoxy granules (<1.0 mm) with the density of 1 g cm^{-3} was used (OISN-1; Applied Ecology Laboratory of Environmental Safety Centre, Odessa, Ukraine). The minimum detectable activities were 0.18 Bq ^{137}Cs and 0.85 Bq ^{241}Am per sample with uncertainties of around 10-15% and 20-30% respectively (p=0.95); sample mass 5 g in petri dish geometry.

Americium-241 and $^{238,239,240}Pu$ activity concentrations in soil samples (*c*. 5 g in Petri dish) were determined using a high purity germanium detector (Canberra GL0520R) with thin beryllium window (energy range of 5 to 700 keV) using the methodology described by Bondarkov et al. [35]. The ^{241}Am activity concentration was estimated using the 59.5 keV gamma emission of its daughter isotope ^{237}Np. The $^{238,239,240}Pu$ activity concentration was estimated using measurement of the L_x-radiation (13-23 keV) emitted from excited uranium daughter isotopes following the α-decay of the Pu-isotopes. Because of absorption of low-energy emissions within the sample, a correction for self-absorption was used. The absorption correction was calculated using the 32-37 keV and 661 keV emissions of ^{137}Ba (^{137}Cs daughter) and assuming the absorption of the 32-37 keV emission was similar to that of U-isotope emissions. The results obtained using this method showed good agreement with those from standard radiochemical methods (±10–15%) for both Pu-isotopes and ^{241}Am for activity concentrations typically found at the most contaminated sites in the CEZ (>10 Bq g^{-1}) [35]. To estimate radionuclide activity concentrations, sample spectra were compared to spectra for ^{238}Pu, ^{239}Pu, ^{240}Pu and ^{241}Am standards. The GL0520R detector was calibrated using a mixed gamma-standard (OISN-343 ^{137}Cs, ^{152}Eu and ^{241}Am epoxy granules (<1.0 mm) with density of 1 g cm^{-3}).

Strontium-90 activity concentrations in soil samples were measured spectrometrically without radiochemical pretreatment; for a detailed description of the method see Bondarkov et al. [36, 37] and Gaschak et al. [38]. The procedure used a β-spectrometer EXPRESS-01 with a thin-film (0.1 mm) plastic scintillator detector calibrated using a ^{90}Sr+^{90}Y standard (OISN-3 expoxy granules <1 mm, density of 1 g cm^{-3}). Daily calibrations of the spectrometer were conducted; uncertainties were approximately 20% (2 sigma).

All equipment, methods and techniques used in the Chornobyl Center laboratory were officially certified and calibrated by State Enterprise ‘KievOblDerzhStandard’ (the state metrological service).

Estimation of absorbed dose rates

We estimated absorbed weighted dose rates for the three relevant soil organisms which are available as defaults within the ERICA Tool version 1.2 [39, 40] and the revised ‘R&D128’ spreadsheet model [41] available from https://wiki.ceh.ac.uk/display/rpemain/Ar+-+Kr+-+Xe+dose+calculator). The ERICA Tool was used to estimate doses to the ‘Annelid’ and ‘Arthropod – detritivorous’ reference organisms, whilst the ‘R&D 128’ spreadsheet model was used to estimate doses for soil bacteria (it is not possible to model bacteria within the ERICA Tool because of limitations on organism sizes). The two models have been shown to give reasonably consistent results for a given organism [42, 43]. To estimate internally incorporated radionuclide concentrations for annelids, and subsequently dose rates, concentration ratios as determined in 2014 for Lumbricidae species collected from a site at the western edge of the Red Forest were used [44]; the concentration ratio is the ratio between the fresh mass activity concentration of the whole body of an organism and the dry mass activity concentration of that radionuclide in soil. For detritivorous arthropods, default concentration ratios from the ERICA Tool were used; there was no need for concentration ratios for bacteria as all exposure is assumed to be external due to their small size. The full set of concentration ratios used in this study is presented in the accompanying dataset [32]. As total Pu activity concentrations in soil were reported, isotopic ratios from Red Forest soil samples collected in 2014 [44] were used to estimate ^{238}Pu, ^{239}Pu and ^{240}Pu activity concentrations for inputting into the ERICA Tool. All three organisms were assumed to have a 100 % occupancy within the soil column and hence a

4π exposure geometry. Measured soil dry mass percentages were used to correct for radiation attenuation within the soil matrix; percentage DM has a proportional influence on the estimated external dose rate, so a 10% soil DM would give an estimated external dose rate of 10% of that if soil were assumed to be 100% dry mass (see discussion in Stark et al. [45]).

Statistical analyses

The Shapiro-Wilk test was used to test for normality of the data prior to subsequent statistical analyses. Tests included, paired t-tests, Kruskal-Wallis test, General Linear Model (GLM) fitting and regression analyses; all tests were performed using Minitab 18. The Red Forest is, in-effect, its own unique habitat, being an area where habitat destruction occurred in 1986. Although there has since been regeneration of deciduous tree species, at the time of this study the Red Forest was generally of poor habitat quality. We have therefore used three simplified habitat classifications for some of our data summarisation and subsequent analyses: 'Red Forest', 'deciduous' and 'coniferous'. Given that soil radionuclide activity concentrations at a given site varied by up to one order of magnitude, we have treated each plot as a separate observation point within our analyses (n=53 plots) rather than averaging across the plots at a given site. Where it was necessary to transform feeding activity data to $\log_e$ values, feeding activity recorded as zero was assumed to be 0.1. For one GLM fitting it was necessary to use R v3.6.1 (see below).

Results and discussion

Soil radionuclide activity concentrations and dose rates

Table 1 summarises radionuclide activity concentrations by simplified habitat (coniferous, deciduous or Red Forest); data for individual sites and plots can be found in Barnett et al. [32]. Plutonium isotope activity concentrations in 17 of the 53 soil samples were below detection

limits; for subsequent dose calculations, Pu-isotope activity concentrations that were below detection limits have been assumed to be the minimum detectable activity concentration.

Table 1. Radionuclide activity concentrations in soils summarised by simple habitat.

	Simple habitat	Number of plots	Arithmetic mean	Arithmetic standard deviation	Minimum	Maximum
**Cs-137 Bq kg^{-1} (DM)**	Coniferous	9	5.76E+03	3.51E+03	2.88E+03	1.40E+04
	Deciduous	20	1.14E+05	1.30E+05	2.84E+03	4.22E+05
	Red Forest	24	4.12E+05	3.32E+05	2.93E+04	1.03E+06
**Sr-90 Bq kg^{-1} (DM)**	Coniferous	9	1.83E+03	6.83E+02	9.00E+02	2.90E+03
	Deciduous	20	9.07E+04	1.73E+05	5.20E+02	7.83E+05
	Red Forest	24	1.68E+05	1.85E+05	1.15E+04	8.66E+05
**Am-241 Bq kg^{-1} (DM)**	Coniferous	9	9.64E+01	2.69E+01	5.40E+01	1.26E+02
	Deciduous	20	5.76E+03	8.58E+03	6.30E+01	3.66E+04
	Red Forest	24	1.20E+04	9.73E+03	1.23E+03	3.24E+04
**Pu-238 Bq kg^{-1} (DM)**	Coniferous	9	5.09E+00	2.44E+00	<3.66E+0	1.14E+01
	Deciduous	20	5.60E+02	7.83E+02	<2.00E+0	2.90E+03
	Red Forest	24	1.87E+03	2.24E+03	9.20E+01	9.49E+03
**Pu-239 Bq kg^{-1} (DM)**	Coniferous	9	9.57E+00	4.59E+00	<6.89E+0	2.14E+01
	Deciduous	20	1.05E+03	1.47E+03	<4.00E+0	5.45E+03
	Red Forest	24	3.52E+03	4.21E+03	1.73E+02	1.79E+04
**Pu-240 Bq kg-1 (DM)**	Coniferous	9	9.57E+00	4.59E+00	<6.89E+0	2.14E+01
	Deciduous	20	1.05E+03	1.47E+03	<4.00E+0	5.45E+03
	Red Forest	24	3.52E+03	4.21E+03	1.73E+02	1.79E+04

Estimated total absorbed dose rates for annelid, detritivorous arthropods and soil bacteria are presented by simple habitat type in Table 2 alongside measured ambient dose rate values. There was a significant relationship between ambient dose rate and the estimated total absorbed dose rates for all three organisms ($p<0.001$; R^2=74-89%). However, in all cases, the ambient dose rate was significantly lower that the total absorbed dose rates ($p<0.001$; paired t-test) (see Table 2); the differences between ambient dose rates and total absorbed dose rates were greatest for the soil bacteria and detritivorous arthropod (for which ^{137}Cs, the dominant component of ambient dose, contributed less to the overall absorbed dose rate than for annelid). Therefore, whilst ambient dose rate is a good marker of comparative external exposure between sites, it

would be erroneous to fit dose-response relationships based on ambient dose rate as others have often done for CEZ dose-effect studies [e.g. 19, 46-48]. For both the annelid and detritivorous arthropod, the largest contributor to absorbed dose was generally ^{137}Cs. However, for detritivorous arthropod ^{241}Am was estimated to contribute a similar percentage of the total dose rate as ^{137}Cs in some cases. The differences between the dose estimated for the annelid and detritivorous arthropod are largely due to the different concentration ratios used to determine organism activity concentrations and consequently the internal dose rate. For annelids we used values derived previously in the Red Forest [44] whereas, for detritivorous arthropod the ERICA Tool (version 1.3) default values [40] were used. With the exception of Pu, the default detritivorous invertebrate concentration ratios were higher than the Red Forest annelid values we have used. The choice of concentration ratio value is acknowledged to be a large contributor to uncertainty in estimated absorbed dose rates for wildlife [49, 50]. However, given the lack of concentration ratio data for detritivorous invertebrates in the CEZ, application of the ERICA Tool default values was necessary.

Table 2. Ambient dose rate and estimated total weighted absorbed dose rate to selected relevant reference organisms summarised by simple habitat type.

	Simple habitat	Number of plots	Arithmetic mean	Arithmetic standard deviation	Minimum	Maximum
**Ambient dose rate (μSv h^{-1})**	Coniferous	9	6.09E-01	5.49E-02	5.00E-01	6.50E-01
	Deciduous	20	2.23E+01	2.63E+01	4.10E-01	7.80E+01
	Red Forest	24	1.01E+02	7.47E+01	1.23E+01	2.37E+02
**Total absorbed dose rate (μGy h^{-1}) Annelid**	Coniferous	9	1.57E+00	8.00E-01	8.40E-01	3.43E+00
	Deciduous	20	4.07E+01	5.00E+01	7.00E-01	1.84E+02
	Red Forest	24	1.45E+02	1.19E+02	1.04E+01	3.89E+02
**Total absorbed dose rate (μGy h^{-1}) Arthropod**	Coniferous	9	2.03E+00	9.12E-01	1.10E+00	4.09E+00
	Deciduous	20	6.15E+01	8.18E+01	1.00E+00	3.30E+02
	Red Forest	24	1.86E+02	1.50E+02	1.46E+01	4.71E+02
**Total absorbed dose rate**	Coniferous	9	4.28E+00	1.27E+00	2.51E+00	6.56E+00
	Deciduous	20	2.16E+02	3.30E+02	2.10E+00	1.41E+03

**(µGy h^{-1})**<br>**Bacteria**	Red Forest	24	6.03E+02	5.28E+02	4.90E+01	1.75E+03

Unsurprisingly, mean absorbed dose rates (and soil activity concentrations) were, highest for the Red Forest (Tables 1 and 2). However, 13 of the 14 plots in deciduous woodland to the south and west of the Red Forest had estimated absorbed dose rates within the range of those estimated for the Red Forest.

The relative difference in estimated total absorbed dose rate for each of the three organisms was broadly consistent across plots. Any differences were due to variation in the isotopic ratios at a given plot. Therefore, in most of the following analyses we present and discuss dose rates for annelids only as conclusions are the same regardless of the organism; annelids have previously been shown to significantly contribute to the observed feeding activity on bait lamina strips [51].

Bait lamina

Although there were some differences in the readings of the bait lamina between the two readers, these were insignificant (p>0.05; paired t-test). Therefore, we have averaged the result of the two readers for use in statistical analyses (individual readings are presented in Barnett et al. [32]). Feeding activity was assessed as qualitative feeding (number of apertures showing any degree of bait consumption (bites)). Utilisation of the bait is summarised by simple habitat type in Table 3. The additional bait lamina strips used to test if inserting into the soil and withdrawing caused notable abrasion showed no damage to the bait.

Table 3. Feeding activity summarised by simple habitat. Feeding activity has been calculated as the total number of bites across the 16 bait lamina strips at each plot (i.e. a

total of 256 apertures per plot). Results are presented for the complete strip (16 apertures per strip) and separately for the top and bottom eight apertures.

	**Simple habitat**	**Number of plots**	**Arithmetic mean**	**Arithmetic standard deviation**	**Minimum**	**Maximum**
**Apertures 1-16 (complete strip)**	Coniferous	9	19.9	9.98	2.00	35.0
	Deciduous	20	20.8	15.6	2.00	53.0
	Red Forest	24	7.73	8.54	0	31.0
**Apertures 1-8 (top of strip)**	Coniferous	9	18.4	9.54	2.00	35.0
	Deciduous	20	15.7	11.6	2.00	45.5
	Red Forest	24	6.31	6.67	0	23.5
**Apertures 9-16 (bottom of strip)**	Coniferous	9	1.44	1.76	0	5.50
	Deciduous	20	5.10	5.39	0	16.5
	Red Forest	24	1.42	2.33	0	7.50

The feeding activity overall was relatively low compared to previous studies conducted elsewhere at sites of differing habitat types (deciduous woodlands, grasslands and arable land) across western Europe not impacted by pollutants [e.g. 26, 27, 52-54] with a mean of 6% of apertures showing evidence of feeding and a maximum for any plot of 21% (the total number of apertures per plot was 256). Four plots, all within the Red Forest, showed no evidence of feeding activity. The bait lamina are known to be an indication of soil faunal activity, with comparatively little contribution of microbial degradation to observed feeding activity [51, 55, 56]. Earthworms have been suggested as contributing significantly to the feeding activity observed on bait lamina sticks [51]. The soils across all of our sites are acidic (pH 3.9 to 4.9) and, in the case of the Red Forest sites and the deciduous woodland sites to the west, generally sandy in nature. Soil conditions such as these are known to result in low earthworm abundance [57] and consequently this is likely to contribute to the low overall utilisation rate of the bait lamina across all sites. The soil temperature at all plots was well above the lower bound of temperatures at which feeding on bait lamina has previously been reported [58].

In agreement with previous studies [e.g. 52-54, 59] utilisation of the bait was highest towards the soil surface (Table 3) with a significant difference in observed feeding activity

between the top eight (*c*. 0.5 to 4 cm below soil surface) and bottom eight (*c*. 4.5 to 8 cm below soil surface) apertures ($p<0.001$; paired t-test). Given the localisation of soil organic matter in the uppermost layers of the soils in the CEZ (S1 and S2 Photographs), the concentration of feeding activity in these upper soil layers is unsurprising.

Estimated absorbed dose rate and feeding activity

There was a significant effect of habitat on feeding activity ($p=0.001$; Kruskal-Wallis test). Median feeding rates in the Red Forest plots were approaching an order of magnitude below those of the deciduous ($p<0.01$) and coniferous ($p<0.05$) woodland plots (note that the bait lamina data were not normally distributed even when transformed to $\log_e$ ($p>0.2$), consequently the GLM fitting was performed using R assuming a Gamma distribution which allows for tails in the data). This difference in median feeding rate might be interpreted as suggesting an effect of radiation exposure on feeding activity. However, a simple linear regression of absorbed dose rate for all three organisms across all 53 plots showed no significant relationship with feeding activity ($p>0.2$; $R^2<0.03$) (Fig 2 presents annelid as an example). Repeating the regression using $\log_e$ transformed total absorbed dose rate and/or feeding activity data did not improve the significance. There was also no relationship if comparisons were restricted to plots within the same simple habitat type. Furthermore, feeding activity at the deciduous woodland sites to the south of the Red Forest, where estimated absorbed dose rates for annelids were in the range 28 – 180 $\mu Gy\ h^{-1}$, were generally high (six of the nine plots being in the upper quartile of all observations) [32]. Conversely, at deciduous woodland sites to the west of the Red Forest, which had dose rates in the range 8 - 21 $\mu Gy\ h^{-1}$, feeding activities were low with ≤5 bites in four of the five plots [32].

Fig 2. A comparison of feeding activity (total bites) and estimated absorbed dose rate for annelid.

Our absorbed dose rate estimates are in-effect an average over the top 10 cm of the soil. However, radionuclide activity concentrations are highest in the upper soil layers [35, 60-62], so the concentration of feeding activity in the upper 4 cm of the soil profile suggests that we may be underestimating the total absorbed dose rates for the organisms with most feeding activity (see Beaugelin-Seiller [63] for discussion of heterogenous radionuclide distribution in soil/sediment profiles).

There are many environmental factors, which affect soil biological activity and which have previously been observed to influence feeding activity as determined using bait lamina strips. These include soil moisture, pH, organic matter content and soil temperature [12, 27; 59]. Whilst organic matter content was not determined for our study soils, bulk density was estimated. Harrison & Bocock [64] present a relationship between surface soil bulk density and organic matter content (the higher the soil bulk density the lower the organic matter content). Consequently, we can assume our estimated soil bulk densities are proxies for organic matter content. Soil pH, percentage moisture, bulk density and temperature values for the study plots are summarised in Table 4; there was no significant difference between April and May soil temperatures ($p>0.3$; paired t-test) and consequently Table 4 presents values averaged across the two measurement times. All measured soil parameters (pH, percentage moisture, bulk density and temperature) show a significant effect of simple habitat ($p<0.003$; Kruskal-Wallis test). Regressions of feeding activity against the soil parameters gave significant relationships for percent moisture ($p<0.001$; $R^2=0.36$) and soil bulk density ($p<0.001$; $R^2=0.40$) (Fig 3); the bulk density relationship in-effect implies that feeding activity increased with increasing soil organic matter content. Whilst the regression of feeding rate against pH was significant, the amount of

variance this explained was poor ($p<0.05$; $R^2<0.09$). There was no significant relationship between feeding rate and soil temperature ($p>0.1$; $R^2=0.04$). Soil temperature may have been in part determined by the time of day when measurements were made; for both the April and May measurements there is a trend in the data suggesting that soil temperature increased with time of day ($p<0.01$; $R^2=0.18$-0.50). However, there was no consistent bias in the time of day at which sites in different habitat classifications were visited. Consequently, we have not considered soil temperature further in our analyses. We note that there is a general lack of soil parameter data available for the CEZ and hence the data from the present study (see [32] and Table 4) makes a valuable contribution.

Table 4. Soil pH, percentage moisture, bulk density and temperature summarised by simple habitat. For a given parameter significant differences ($p<0.05$; generalised linear model) between habitats are identified by different superscripted letter.

	Simple habitat	Number of plots	Arithmetic mean	Arithmetic standard deviation	Minimum	Maximum
**pH**	Coniferous	9	3.87[a]	0.10	3.74	4.05
	Deciduous	20	4.33[a]	0.52	3.52	5.08
	Red Forest	24	4.58[b]	0.25	3.94	4.93
**% moisture**	Coniferous	9	19.3[a]	5.17	10.6	26.4
	Deciduous	20	23.7[a]	12.7	7.07	49.6
	Red Forest	24	9.43[b]	2.06	4.40	13.6
**Soil bulk density (g cm^{-3})**	Coniferous	9	0.78[a]	0.13	0.57	0.92
	Deciduous	20	0.86[a]	0.30	0.39	1.38
	Red Forest	24	1.24[b]	0.08	1.10	1.39
**Soil temperature (^{o}C)**	Coniferous	9	9.52[a]	0.26	9.05	9.90
	Deciduous	20	9.68[a]	0.81	8.15	11.5
	Red Forest	24	10.8[b]	1.57	9.35	15.5

Fig 3. Relationship between feeding activity (total bites) and soil percentage moisture (top) and soil bulk density (bottom).

Given there was an effect of habitat/soil parameters on the feeding activity we observed, we have conducted regression analyses of feeding activity against absorbed dose rate with soil moisture, soil pH and soil bulk density included as continuous predictors. The resultant statistics (presented for the example of annelid in Table 5) demonstrate no significant interaction between feeding rate and dose rate when these other variables were taken into account. A lack of relationship between feeding activity and absorbed dose rate was also found for the detritivorous arthropod and soil bacteria ($p>0.2$); by estimating the absorbed dose rates for the smallest (bacteria) and largest soil organisms (annelids) we should have encompassed the range of likely dose rates to any organism feeding on the bait lamina (e.g. nematodes, collembola, mites [65]). We repeated the analyses without the soil parameters, but with simple habitat as a categorical predictor. Again, there was no significant influence of absorbed dose rate on feeding activity ($p>0.3$ in the case of the annelid).

Table 5. Statistics of regression for annelid.

	DF	Adj SS	Adj MS	F-Value	P-Value
**Regression**	45	6.32E+03	1.40E+02	0.33	0.988
**Total absorbed dose rate ($\mu Gy\ h^{-1}$) Annelid**	1	2.62E+01	2.62E+01	0.06	0.810
**pH**	44	6.06E+03	1.38E+02	0.33	0.989
**Error**	7	2.94E+03	4.20E+02		
**Total**	52	9.26E+03			

Conclusions

Our study indicates no effect of current radiation exposure on soil biological activity as determined by bait lamina strips. This is in agreement with the leaf litter decomposition study of Bonzom et al. [20] but disagrees with the conclusion of Mousseau et al. [19] that '*we have shown severely depressed levels of litter mass loss in the most contaminated forest areas around Chernobyl*' (sic). A criticism of the Bonzom et al. study could be that it considered a lower dose

rate range than the work of Mousseau et al.. However, the maximum ambient dose rate across our sites was comparable to that quoted by Mousseau et al.. Furthermore, from the map of study sites presented in Mousseau et al. and their supplementary data table, it would appear that a number of their sites were in similar locations to those used in our study.

The study presented here is the largest deployment of bait lamina reported to date within the CEZ. To our knowledge bait lamina have been used in the CEZ twice before in limited scoping studies. Across four sites Jackson et al. [66] found a decreasing trend in bait lamina utilisation with increasing ambient dose rate (gamma air kerma ranged from 0.1-0.5 μGy h^{-1} to 60-138 μGy h^{-1}), the lowest feeding activity being observed in a site referred to as the Red Forest (the Jackson et al. study was conducted in 2002). Conversely, unreported data by some of the authors of this paper shows no relationship between feeding activity and soil ^{137}Cs (range 3–140 kBq kg^{-1} DM) or ^{90}Sr (range 2–150 kBq kg^{-1} DM) activity concentrations across four CEZ sites (including two towards the western end of the Red Forest) in summer 2005. As these data are unpublished, we have included them within the dataset accompanying this paper [32] to enable independent consideration.

We acknowledge that the endpoint of bait lamina is a measure of invertebrate feeding activity whereas litter bags, used by both Mousseau et al. [19]) and Bonzom et al. [20], give an estimation of organic matter decomposition [67] and consequently the two methods likely predominantly study different organisms. Comparisons between the two approaches differ, with some authors observing similar trends in the results of the two approaches [67-69) and others finding different trends [70, 71). However, Mousseau et al. [19] imply that their results demonstrated a similar impact of radiation on decomposition by microbial communities and by detritivorous invertebrates (assessed by comparing the results from fine and coarse mesh litter bags respectively). In future studies, it is important that authors do not extrapolate their findings beyond the limits of the method adopted. A more holistic evaluation of the influence of radiation

on soil surface and sub-surface biological activity would be obtained by combining litter decomposition studies with bait lamina deployment.

Rather than current chronic radiation dose rates, soil biological activity varies with soil properties (organic matter content (inferred from soil bulk density), moisture content and pH). These are all well known to influence soil biological activity and feeding activity as determined using bait lamina strips (e.g. [12, 27). Our finding is in contrast to that of Mousseau et al. [19] who state that their litter bag decomposition data showed a linear dose response of decomposition independent of confounding variables such as pH and soil moisture. The analyses of Mousseau et al. did not appear to consider soil organic matter content as a variable. However, they state that they observed an accumulation of litter with increasing radiation. This is in disagreement with our findings that, irrespective of dose rate, soil biological activity likely increased with increasing soil organic matter content (as inferred for soil bulk density measurements). Furthermore, as noted above, our sites in the Red Forest visually had a sparse litter layer and the most contaminated sites of Mousseau et al. must also have been in the Red Forest (based upon their sample site map). Although our litter layer observation is anecdotal, and cannot be verified, it is supported by our soil bulk density measurements (Table 4).

Whilst we found no relationship between soil invertebrate feeding activity and absorbed dose rate, this does not necessarily mean that radiation has had no impact on soil biota within the CEZ. When analysed by simple habitat category, the Red Forest showed significantly lower feeding activity than the deciduous or coniferous plots. As discussed in Beresford et al. [72] the Red Forest is a unique habitat which was altered by radiation in 1986 and which, at the time of the work reported in this paper, continued to have a relatively poor habitat status. In a review of the impacts of the 1957 Kyshtym (Russian Urals) accident, Fesenko [73] reports that soil invertebrate communities had not been restored at a contaminated site (the main contaminant being ^{90}Sr) *c*. 30 years after the accident. Fesenko suggested that, in part, continued impacts on

soil invertebrates was due to their low mobility and hence a lack of migration into the area. As soil invertebrates have low dispersal rates (e.g. of the order of 5-10 m a^{-1} for earthworms [74]) such a long-term impact of an acute radiation event would seem plausible. Krivolutzkii & Pokarzhevskii [16] report that young earthworms did not survive or hatch in autumn of 1986 close to the ChNPP due to their greater radiosensitivity compared to adults.

We have estimated total absorbed dose rates for relevant organism types rather than simply using ambient dose rate as a marker of comparative radiation levels across our study sites. In agreement with previous observations [44, 75, 76] we demonstrated that ambient dose rate underestimated total absorbed dose rates to organisms. We recommend that, when relating observations to radiation exposure, total absorbed dose rates are used. This is not a new suggestion (e.g. see Chesser & Baker [77]) but unfortunately bad practice often seems to persist.

There are two aspects of our paper which we would like to draw attention to and encourage as good practice. Firstly, the bait lamina sticks were read ‘blind’ by people with no knowledge of where they had been deployed (again ‘blind’ analysis has been recommended previously for studies in the CEZ by Chesser & Baker [77]). Where possible (and it is obviously not possible for observations/measurements made by researchers in the field) blind analysis should be used in future studies as it reduces the potential for bias, either unintentional or intentional, and minimises future criticism. Secondly, there is considerable debate in the scientific literature about the long-term impacts of chronic exposure of wildlife to radiation in the Chernobyl Exclusion Zone and now also in the Fukushima impacted areas (see Beresford et al. [78]). This lack of consensus has a relatively high public profile, with potential impacts on, for instance, the use of radiation (from medicine to nuclear power) and strategies for remediating areas contaminated by nuclear accidents. By publishing the complete underlying data for our paper [32], we give other scientists the ability to confirm our conclusions or indeed refute them should that be valid. Such an approach of open data publication is the norm in some scientific areas (e.g. for sequencing

data, https://www.ncbi.nlm.nih.gov/sra). A wider willingness to make data freely available in this way would greatly aid the scientific community reaching much needed consensus on the effects of radiation on wildlife under field conditions.

Acknowledgements

The authors would like to thank Claire Wells (UKCEH) for reading the bait lamina strips, Peter Henrys (UKCEH) for statistical advice, Jacky Chaplow (UKCEH) for help in preparing the accompanying published dataset, Andrea Maksimenko (Chornobyl Center) for radioanalyses and Eugene Guliaichenko (Chornobyl Center) for assistance during sample preparation and fieldwork.

References

1. Weil RR, Brady NC. The nature and properties of soils. 15th ed. Columbus: Pearson; 2017.
2. Morgan JE, Morgan AJ. Earthworms as biological monitors of cadmium, copper, lead and zinc in metalliferous soils. Environ Pollut 1998;54: 123-138. doi: 10.1016/0269-7491(88)90142-X
3. van Gestel CA, van der Waarde JJ, Derksen JG, van der Hoek EE, Veul MF, Bouwens S. et al. The use of acute and chronic bioassays to determine the ecological risk and bioremediation efficiency of oil- polluted soils. Environ Toxicol Chem 2001;20: 1438-1449. doi: 10.1002/etc.5620200705
4. Spurgeon DJ, Weeks JM, Van Gestel, CAM. A summary of eleven years progress in earthworm ecotoxicology. Pedobiologia 2003;47 588-606. doi: 10.1078/0031-4056-00234

5. Thomsen M, Faber JH, Sorensen PB. 2012. Soil ecosystem health and services - Evaluation of ecological indicators susceptible to chemical stressors. Ecol Indic 2012;16: 67-75. doi: 10.1016/j.ecolind.2011.05.012
6. Jones OA, Sdepanian S, Lofts S, Svendsen C, Spurgeon DJ, Maguire ML. et al. Metabolomic analysis of soil communities can be used for pollution assessment. Environ Toxicol Chem 2014;33: 61-64. doi: 10.1002/etc.2418
7. Römbke J. The feeding activity of invertebrates as a functional indicator in soil. Plant Soil 2014;383: 43-46. doi: 10.1007/s11104-014-2195-5
8. Gunstone T, Cornelisse T, Klein K, Dubey A, Donley N. Pesticides and soil invertebrates: A hazard assessment. Front Environ Sci 2021;9: 122. doi: 10.3389/fenvs.2021.643847
9. Zaitsev AS, Gongalsky KB, Nakamori T, Kaneko N. Ionizing radiation effects on soil biota: Application of lessons learned from Chernobyl accident for radioecological monitoring. Pedobiologia 2013;57: 5-14. doi: 10.1016/j.pedobi.2013.09.005
10. United Nations Scientific Committee on the Effects of Atomic Radiation (UNSCEAR). Sources and effects of ionizing radiation. Report to the General Assembly with Scientific Annex. New York: United Nations; 1996.
11. Andersson P, Garnier-Laplace J, Beresford NA, Copplestone D, Howard BJ, Howe P. et al. Protection of the environment from ionising radiation in a regulatory context (PROTECT): proposed numerical benchmark values. J Environ Radioact 2009;100: 1100-1108. doi: 10.1016/j.jenvrad.2009.05.010
12. André A, Antunes SC, Gonçalves F, Pereira R. Bait-lamina assay as a tool to assess the effects of metal contamination in the feeding activity of soil invertebrates within a uranium mine area. Environ Pollut 2009;157: 2368-2377. doi: 10.1016/j.envpol.2009.03.023

13. Gongalsky KB. Impact of pollution caused by uranium production on soil macrofauna. Environ Monit Assess 2003;89: 197-219. doi: 10.1023/A:1026031224658

14. Haanes H, Gjelsvik R. Reduced soil fauna decomposition in a high background radiation area. PLOS ONE 2021;16: e0247793. doi: 10.1371/journal.pone.0247793

15. Rogiers T, Claesen J, Van Gompel A, Vanhoudt N, Mysara M, Williamson A, Leys, N. et al. Soil microbial community structure and functionality changes in response to long-term metal and radionuclide pollution. Environ Microbiol 2021;23: 1670-1683. doi: 10.1111/1462-2920.15394

16. Krivolutzkii DA, Pokarzhevskii AD. Effects of radioactive fallout on soil animal populations in the 30 km zone of the Chernobyl atomic power station. Sci Tot Environ 1992;12: 69-77. doi: 10.1016/0048-9697(92)90239-O

17. Geras’kin SA, Fesenko SV, Alexakhin RM. Effects of non-human species irradiation after the Chernobyl NPP accident. Environ Int 2008;34: 880-897. doi: 10.1016/j.envint.2007.12.012

18. Beresford NA, Barnett CL, Gashchak S, Kashparov V, Kireev S, Levchuk S. et al. Wildfires in the Chernobyl Exclusion Zone – risks, consequences and management. Integr Environ Assess Manag 2021. doi: 10.1002/ieam.4424

19. Mousseau T, Milinevsky G, Kenney‐ Hunt J, Møller A. Highly reduced mass loss rates and increased litter layer in radioactively contaminated areas. Oecologia 2014;175: 429-437. doi: 10.1007/s00442-014-2908-8

20. Bonzom J-M, Hättenschwiler S, Lecomte-Pradines C, Chauvet E, Gaschak S, Beaugelin-Seiller K. et al. Effects of radionuclide contamination on leaf litter decomposition in the Chernobyl exclusion zone. Sci Tot Environ 2016;562: 596-603. doi: 10.1016/j.scitotenv.2016.04.006

21. Chapon V, Piette, L, Vesvres M-H, Coppin F, Le Marrec C, Christen R. Microbial diversity in contaminated soils along the T22 trench of the Chernobyl experimental platform. Appl Geochem 2012;27: 1375-1383. doi: 10.1016/j.apgeochem.2011.08.011

22. Jones HE. Impact of anthropogenic ionising radiation on soil microbial communities. PhD thesis, University of Nottingham; 2004.

23. Lecomte-Pradines C, Bonzom J.-M, Della-Vedova C, Beaugelin-Seiller K, Villenave C, Gaschak S. et al. Soil nematode assemblages as bioindicators of radiation impact in the Chernobyl Exclusion Zone. Sci Tot Environ 2014;490: 161-170. doi: 10.1016/j.scitotenv.2014.04.115

24. Newbold LK., Robinson A. Rasnaca I, Lahive E, Soon GH, Lapied E, Oughton D, Gashchak S, Beresford NA, Spurgeon DJ. Genetic, epigenetic and microbiome characterisation of an earthworm species (*Octolasion lacteum*) along a radiation exposure gradient at Chernobyl. Environ Pollut 2019;255, 113238. doi: 10.1016/j.envpol.2019.113238

25. Kratz W. 1998. The bait-lamina test - General aspects, applications and perspectives. Environ Sci Pollut Res 1998;5: 94-96. doi: 10.1007/BF02986394

26. Förster B, Van Gestel CAM, Koolhaas JEE, Nentwig G, Rodrigues JML, Sousa JP. et al. Ring-testing and field-validation of a terrestrial model ecosystem (TME) - an instrument for testing potentially harmful substances: effects of carbendazim on organic matter breakdown and soil fauna feeding activity. Ecotoxicology 2004;13: 129-141. doi: 10.1023/B:ECTX.0000012410.99020.97

27. Boshoff M, De Jonge M, Dardenne F, Blust R, Bervoets L. The impact of metal pollution on soil faunal and microbial activity in two grassland ecosystems. Environ Res 2014;134: 169-180. doi: 10.1016/j.envres.2014.06.024.

28. Jänsch S, Scheffczyk A, Römbke J. The bait-lamina earthworm test: a possible addition to the chronic earthworm toxicity test? Euro-Mediterr. J Environ Integr 2017;2: 5. doi: 10.1007/s41207-017-0015-z

29. Bispo A, Cluzeau D, Creamer R, Dombos M, Graefe U, Krogh PH. et al. Indicators for monitoring soil biodiversity. Integr Environ Assess Manag 2009;5: 717-719. doi: 10.1897/IEAM_2009-064.1

30. Environment Agency (EA). Guidance on the use of bioassays in Ecological Risk Assessment. SCO70009/SR2c. Bristol: Environment Agency; 2008.

31. International Organization for Standardization (ISO). Soil quality - Method for testing effects of soil contaminants on the feeding activity of soil dwelling organisms - Bait-lamina test. ISO 18311:2016. Geneva: ISO; 2016. Available from: https://www.iso.org/standard/62102.html

32. Barnett CL, Gashchak S, Wells, C, Maksimenko A, Chaplow J, Wood MD, Parekh NR, Beresford NA. Soil biological activity in the Chernobyl Exclusion Zone, Ukraine, September 2006 and spring 2016. NERC Environmental Information Data Centre. (Dataset). 2021. doi: 10.5285/19babe1c-b3a3-488c-b4fe-ebb4ab9237d8

33. Smith J, Beresford NA. Chernobyl Catastrophe and Consequences. Chichester: Praxis Publishing/Springer; 2005. Available from: https://link.springer.com/book/10.1007/3-540-28079-0

34. Allen SE. Chemical analysis of ecological materials. Oxford: Blackwell Scientific Publications; 1974.

35. Bondarkov MD, Zheltonozhsky VA, Zheltonozhskaya MV, Kulich NV, Maksimenk AM, Farfán EB, et al. Vertical migration of radionuclides in the vicinity of the Chernobyl confinement shelter. Hlth Phys 2011a;101: 362-367. doi: 10.1097/HP.0b013e3182166472

36. Bondarkov MD, Bondarkov DM, Zheltonozhsky VA, Maksimenko AM, Sadovnikov LV, Strilchuk NV. A method of 90Sr concentration measurement in biological objects and soil samples without radiochemistry. Nucl Phys At Energy 2002;2: 162-167. Russian.

37. Bondarkov MD, Maksimenko AM, Gaschak S, Zheltonozhsky VA, Jannik GT, Farfán EB. Method for simultaneous ^{90}Sr and ^{137}Cs in-vivo measurements of small animals and other environmental media developed for the conditions of the Chernobyl exclusion zone. Hlth Phys 2011b;101: 383-392. doi: 10.1097/HP.0b013e318224bb2b

38. Gaschak SP, Makliuk YA, Maksimenko AM, Bondarkov MD, Chizhevsky I, Caldwell EF. et al. Frequency distributions of ^{90}Sr and ^{137}Cs concentrations in an ecosystem of the 'Red Forest' area in the Chernobyl Exclusion Zone. Hlth Phys 2011;101: 409-415. doi: 10.1097/HP.0b013e31821d0b81

39. Brown JE, Alfonso B, Avila R, Beresford NA, Copplestone D, Pröhl G. et al. The ERICA Tool. J Environ Radioact 2008;99: 1371-1383. doi: 10.1016/j.jenvrad.2008.01.008

40. Brown JE, Alfonso B, Avila R, Beresford NA, Copplestone D, Hosseini A. A new version of the ERICA Tool to facilitate impact assessments of radioactivity on wild plants and animals. J Environ Radioact 2016;53: 141-148. doi: 10.1016/j.jenvrad.2015.12.011

41. Copplestone D, Bielby S, Jones SR, Patton D, Daniel P, Gize I. Impact assessment of ionising radiation on wildlife. R&D Publication 128. Bristol: Environment Agency; 2001.

42. Vives i Batlle J, Balonov M, Beaugelin-Seiller K, Beresford NA, Brown J, Cheng J-J. et al. Inter-comparison of absorbed dose rates for non-human biota. Radiat Environ Biophys 2007;46 349-373. doi: 10.1007/s00411-007-0124-1

43. Vives i Batlle J, Beaugelin-Seiller K, Beresford NA, Copplestone D, Horyna J. Hosseini A. et al. The estimation of absorbed dose rates for non-human biota: an extended intercomparison. Radiat Environ Biophys 2011;50: 231-251. doi: 10.1007/s00411-010-0346-5

44. Beresford NA, Barnett CL, Gashchak S, Maksimenko A, Guliaichenko E, Wood MD. et al. Radionuclide transfer to wildlife at a 'Reference Site' in the Chernobyl Exclusion Zone and resultant radiation exposures. J Environ Radioact 2020a;211: 105661. doi: 10.1016/j.jenvrad.2018.02.007

45. Stark K, Andersson P, Beresford NA, Yankovich TL, Wood MD, Johansen MP. et al. Predicting exposure of wildlife in radionuclide contaminated wetland ecosystems. Environ Pol 2015;196: 201-213. doi: 10.1016/j.envpol.2014.10.012

46. Møller AP, Mousseau TA. Reduced abundance of insects and spiders linked to radiation at Chernobyl 20 years after the accident. Biol Lett 2009;5: 356-359. doi: 10.1098/rsbl.2008.0778

47. Møller AP, Mousseau TA. Assessing effects of radiation on abundance of mammals and predator–prey interactions in Chernobyl using tracks in the snow. Ecol Indicat 2013;26 112-116. doi: 10.1016/j.ecolind.2012.10.025

48. Lehmann P, Boratyński Z, Mappes T, Mousseau TA, Møller AP. Fitness costs of increased cataract frequency and cumulative radiation dose in natural mammalian populations from Chernobyl. Sci Rep 2016;6: 19974. doi: 10.1038/srep19974

49. Beresford NA, Balonov M, Beaugelin-Seiller K, Brown J, Copplestone D, Hingston JL. et al. An international comparison of models and approaches for the estimation of the radiological exposure of non-human biota. Int J Rad Appl Instrum A 2008;66: 1745-1749. doi: 10.1016/j.apradiso.2008.04.009

50. Robinson CA, Smith KL, Norris S. Impacts on non-human biota from a generic geological disposal facility for radioactive waste: some key assessment issues. J Radiol Prot 2010;30: 161-173. doi: 10.1088/0952-4746/30/2/005

51. Hamel C, Schellenberg MP, Hanson K, Wang H. Evaluation of the “bait-lamina test” to assess soil microfauna feeding activity in mixed grassland. Appl Soil Ecol 2007;36: 199-204. doi: 10.1016/j.apsoil.2007.02.004

52. Geissen V, Brümmer G. Decomposition rates and feeding activities of soil fauna in deciduous forest soils in relation to soil chemical parameters following liming and fertilization. Biol Fertil Soils 1999;29: 335-342. doi: 10.1007/s003740050562

53. Filzek PDB, Spurgeon DJ, Broll G, Svendsen C, Hankard PK, Parekh N. et al. Metal effects on soil invertebrate feeding: measurements using the bait lamina method. Ecotoxicology 2004;13: 807-816. doi: 10.1007/s10646-003-4478-0

54. Riutta T, Clack H, Crockatt M, Slade EM. Landscape-scale implications of the edge effect on soil fauna activity in a temperate forest. Ecosystems 2016;19: 534-544. doi: 10.1007/s10021-015-9939-9

55. Domene X, Mattana S, Hanley, K, Enders, A, Lehmann J. Medium-term effects of corn biochar addition on soil biota activities and functions in a temperate soil cropped to corn. Soil Biol Biochem 2014;72: 152-162. doi: 10.1016/j.soilbio.2014.01.035

56. Helling B, Pfeiff G, Larink O. A comparison of feeding activity of collembolan and enchytraeid in laboratory studies using the bait-lamina test. Appl Soil Ecol 1998;7: 207-212. doi: 10.1016/S0929-1393(97)00065-6

57. Curry JP. Factors affecting the abundance of earthworms in soils. 2nd ed. In: Edwards C, editor. Earthworm Ecology. Boca Raton: CRC Press; 2004. pp 91-114.

58. Gongalsky KB, Persson T, Pokarzhevskii AD. Effects of soil temperature and moisture on the feeding activity of soil animals as determined by the bait-lamina test. Appl Soil Ecol 2008;39: 84-90. doi: 10.1016/j.apsoil.2007.11.007

59. Simpson JE, Slade E, Riutta T, Taylor ME. Factors affecting soil fauna feeding activity in a fragmented lowland temperate deciduous woodland. PLOS ONE 2012;7: e29616. doi: 10.1371/journal.pone.0029616

60. Holiaka DM, Levchuk SE, Kashparov, VA, Holiaka MA, Yoschenko LV, Otreshko LN. et al. Vertical distribution of ^{90}Sr in soil profiles and its uptake by scots pine (*Pinus Sylvestris* L.) wood growing within the Chernobyl exclusion zone. Nucl Phys At Energy 2020a;21: 157-165. Ukranian. doi: 10.15407/jnpae2020.02.157

61. Holiaka DM, Levchuk SE, Yoschenko VI, Kashparov VA, Yoschenko LV, Holiaka MA, et al. ^{90}Sr and ^{137}Cs inventories in the depots and biogenic fluxes of the typical forest stands in the Chernobyl exclusion zone. Nucl Phys At Energy 2020b;21: 256-264. Ukranian. doi: 10.15407/jnpae2020.03.256

62. Levchuk SE, Holiaka DM, Pavlyuchenko VV. Radionuclide distribution down soil profiles in the Chernobyl Exclusion Zone 2020. NERC Environmental Information Data Centre. (Dataset). doi: 10.5285/72c05810-eb66-49e1-9107-dec8cde3b07e

63. Beaugelin-Seiller K. The assumption of heterogeneous or homogeneous radioactive contamination in soil/sediment: does it matter in terms of the external exposure of fauna? J Environ Radioact 2014;138: 60-67. doi: 10.1016/j.jenvrad.2014.07.027

64. Harrison AF, Bocock KL. Estimation of soil bulk-density from loss-on-ignition values. J Appl Ecol 1981;18: 919-927. doi: 10.2307/2402382

65. Siebert J, Sünnemann M, Auge H, Berger S, Cesarz M, Ciobanu N. The effects of drought and nutrient addition on soil organisms vary across taxonomic groups, but are constant across seasons. Sci Rep 2019;9: 639. doi: 10.1038/s41598-018-36777-3

66. Jackson D, Copplestone D, Stone DM, Smith. Terrestrial invertebrate population studies in the Chernobyl exclusion zone, Ukraine. Radioprotection 2005;40: S857-S863. doi: 10.1051/radiopro:2005s1-126

67. van Gestel CAM, Koolhaas JE, Hamers T, van Hoppe M, van Roovert M, Korsman C. et al. Effects of metal pollution on earthworm communities in a contaminated floodplain area: Linking biomarker, community and functional responses. Environ Pollut 2009;157: 895-903. doi: 10.1016/j.envpol.2008.11.002

68. Mbodj I, Sarr M, Diarra K. Using bait lamina and litterbags, two functional methods to monitor biological activity in soil contaminated by dieldrin. Preliminary results from Dakar (Senegal) sahelian region. Int J Biol Chem Sci 2010;4: 122-129. doi: 10.4314/ijbcs.v4i1.54238

69. Walter J, Hein R, Beierkuhnlein C, Hammerl V, Jentsch A, Schädler M. et al. Combined effects of multifactor climate change and land-use on decomposition in temperate grassland. Soil Biol Biochem 2013;60 10-18. doi: 10.1016/j.soilbio.2013.01.018

70. Paulus R, Römbke J, Ruf A, Beck L. A comparison of the litterbag-, minicontainer-, and bait-lamina-methods in an ecotoxicological field experiment with diflubenzuron and Btk. Pedobiologia 1999;43: 120-133.

71. Menezes- Oliveira VB, Scott- Fordsmand JJ, Soares AM, Amorim MJ. Effects of temperature and copper pollution on soil community-extreme temperature events can lead to community extinction. Environ Toxicol Chem 2013;32: 2678-2685. doi: 10.1002/etc.2345

72. Beresford NA, Scott EM, Copplestone D. Field effects studies in the Chernobyl Exclusion Zone: Lessons to be learnt. J Environ Radioact 2020b;211: 105893. doi: 10.1016/j.jenvrad.2019.01.005

73. Fesenko S. Review of radiation effects in non-human species in areas affected by the Kyshtym accident. J Radiol Prot 2019;39: R1-R17. doi: 10.1088/1361-6498/aafa92

74. Marinissen JCY, van den Bosch F. Colonization of new habitats by earthworms. Oecologia 1992;91: 371-376. doi: 10.1007/BF00317626

75. Antwis RE, Beresford NA, Jackson JA, Fawkes R, Barnett CL, Potter E. et al. Impacts of radiation on the bacterial and fungal microbiome of small mammals in the Chernobyl Exclusion Zone. J Anim Ecol 2021. doi:10.1111/1365-2656.13507

76. Beaugelin-Seiller K, Garnier-Laplace J, Beresford NA. Estimating radiological exposure of wildlife in the field. J Environ Radioact 2020;211: 105830. doi: 10.1016/j.jenvrad.2018.10.006

77. Chesser RK, Baker RJ. Growing up with Chernobyl. Working in a radioactive zone, two scientists learn tough lessons about politics, bias and the challenges of doing good science. Am Scientists 2006; 94: 542-549. doi: https://www.jstor.org/stable/27858869

78. Beresford NA, Horemans N, Copplestone D, Raines KE, Orizaola G, Wood MD. et al. Towards solving a scientific controversy - The effects of ionising radiation on the environment. J Environ Radioact 2020c;211: 106033. doi: 10.1016/j.jenvrad.2019.106033

Supporting information

S1 Photograph. Example profile for the soil CEZ - Plot 18.1 (see Barnett et al. [32]) showing a profile typical for much of the CEZ (including the Red Forest) with little visible organic matter.

S2 Photograph. Example profile for the soil CEZ - Plot 2.1 (see Barnett et al. [32]) soil profile for site in the deciduous woodland to the south of the Red Forest showing a defined organic matter layer.

Figure 1

Click here to access/download;Figure;Fig 1.tif

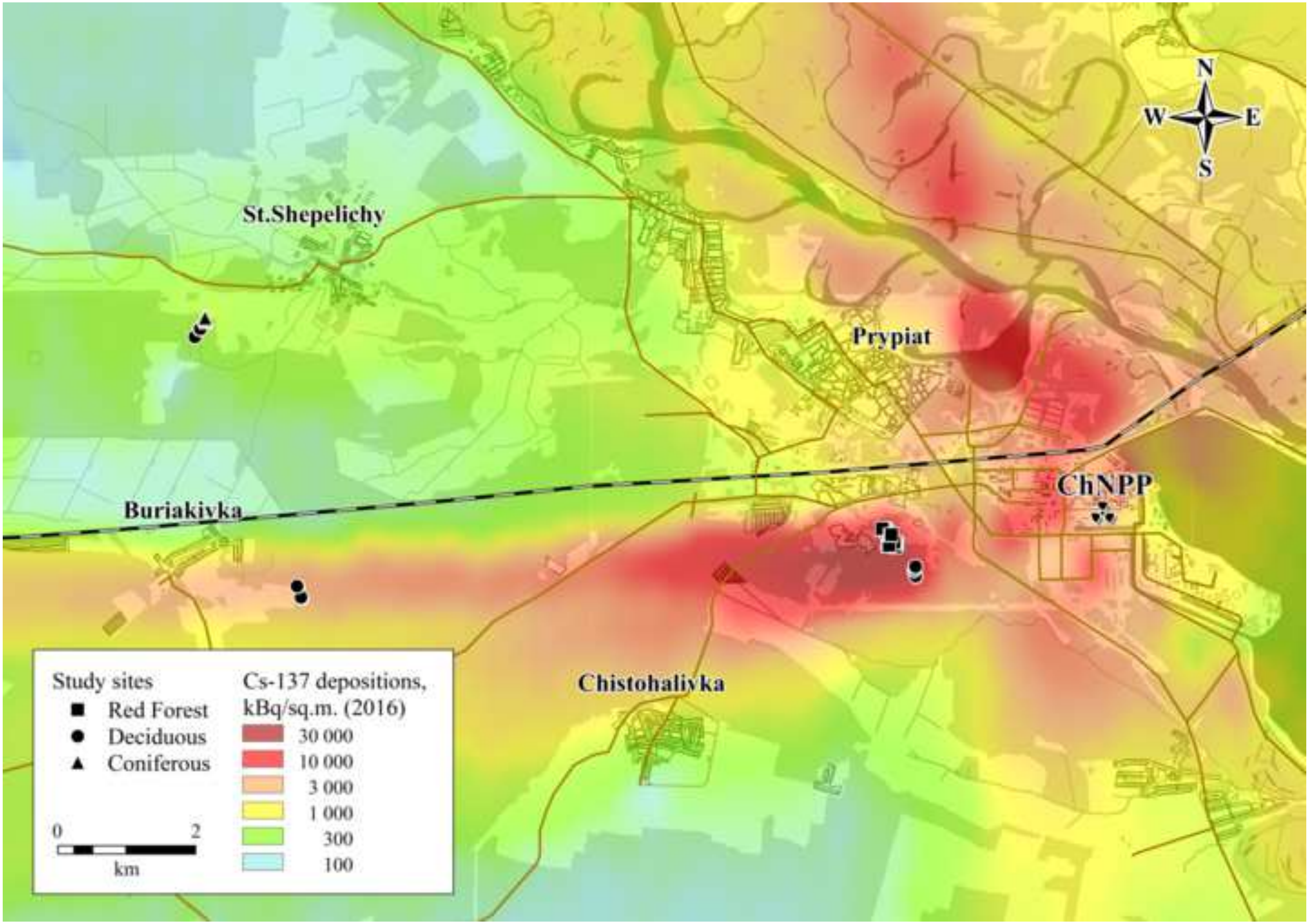

Figure 2

Click here to access/download;Figure;Fig 2.tif

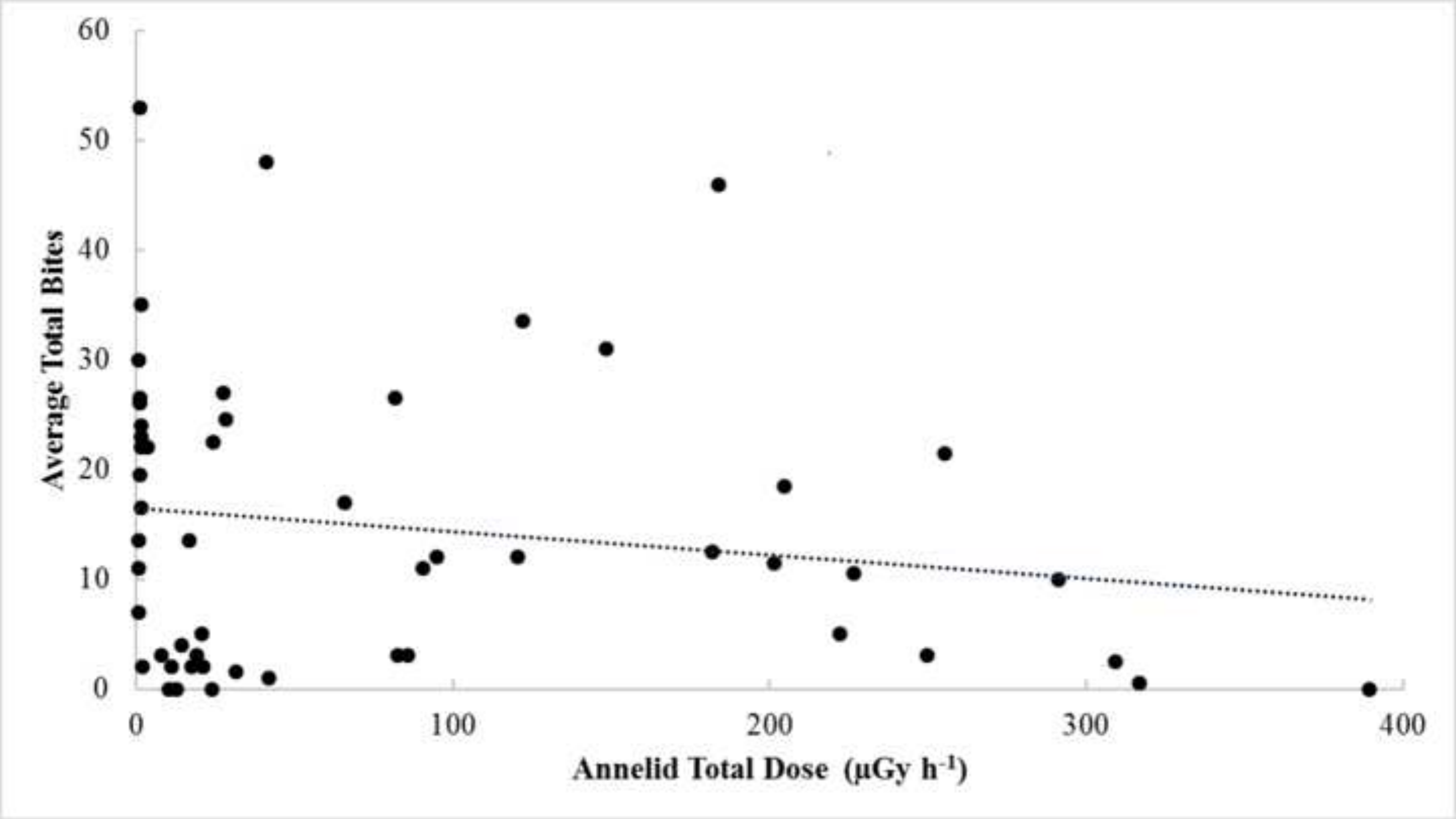

Figure 3

Click here to access/download;Figure;Fig 3.tif

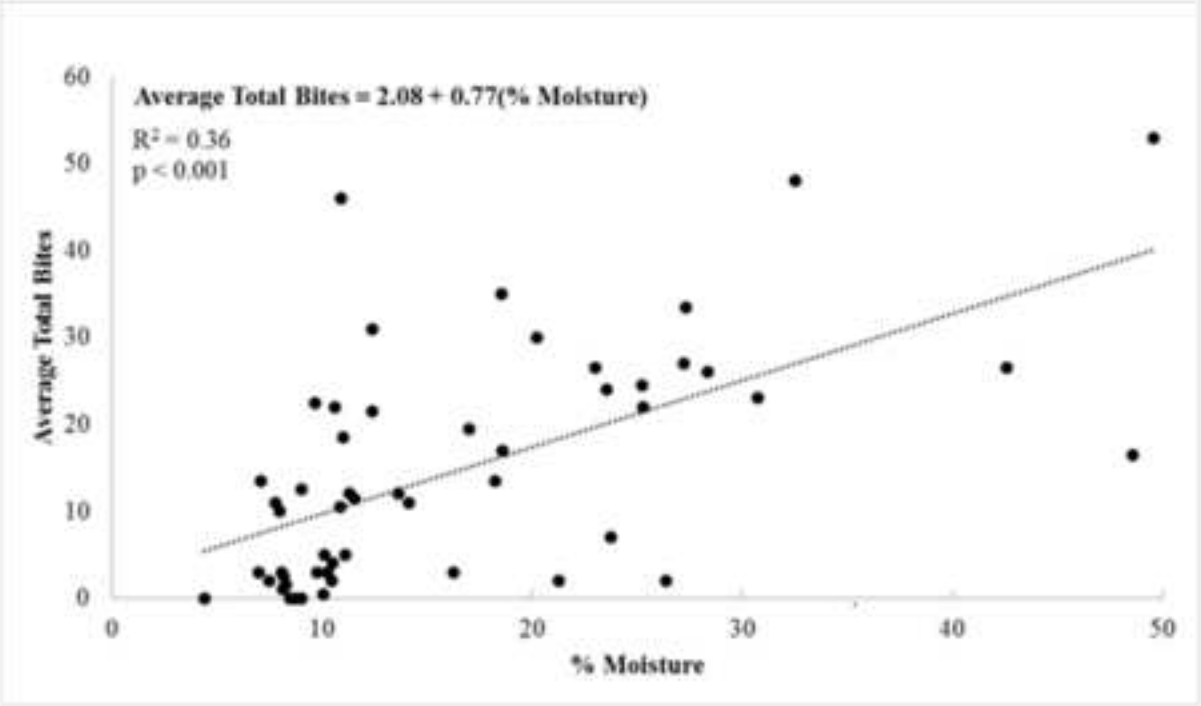

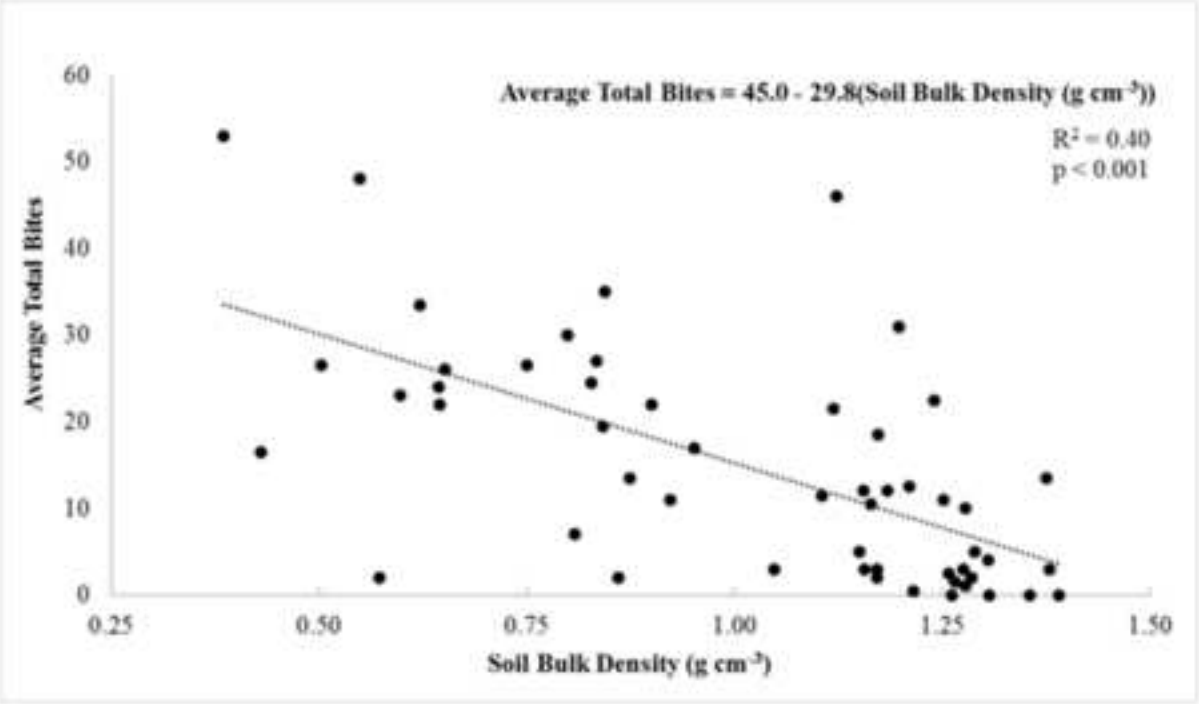

Photograph S1

Click here to access/download
Supporting Information
S1 Photograph.jpg

Photograph S2

Click here to access/download
Supporting Information
S2 Photograph.jpg
